# *trans*-Diaqua­bis­(1*H* imidazolium-4,5-di­carboxyl­ato-κ^2^ *O* ^4^,*O* ^5^)magnesium

**DOI:** 10.1107/S1600536812017667

**Published:** 2012-04-28

**Authors:** Katarzyna Łuczyńska-Szymczak, Wojciech Starosta, Janusz Leciejewicz

**Affiliations:** a Institute of Nuclear Chemistry and Technology, ul.Dorodna 16, 03-195 Warszawa, Poland

## Abstract

The title compound, [Mg(C_5_H_3_N_2_O_4_)_2_(H_2_O)_2_], consists of centrosymmetric neutral monomers in which two *O*,*O*′-bidentate imidazolinium-4,5-dicarboxyl­ate ligands are bonded to the Mg^II^ ion. One of the carboxyl protons is transferred to the N atom of the imidazole ring. The octa­hedral metal-ion coordination is completed by two *trans* water O atoms. In the crystal, mol­ecules are linked by N—H⋯(O,O) and O—H⋯O hydrogen bonds.

## Related literature

For the crystal structures of two Ca^II^ complexes with imidazole-4,5-dicarboxyl­ate and aqua ligands, see: Starosta *et al.* (2006 ▶) and for the structure of a Ba^II^ complex, see: Starosta *et al.* (2007 ▶).
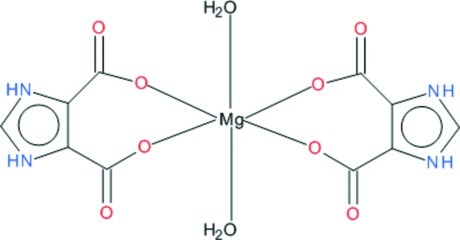


## Experimental

### 

#### Crystal data

[Mg(C_5_H_3_N_2_O_4_)_2_(H_2_O)_2_]
*M* *_r_* = 370.53
Monoclinic, 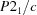

*a* = 7.2545 (15) Å
*b* = 13.847 (3) Å
*c* = 6.9975 (14) Å
β = 115.46 (3)°
*V* = 634.6 (2) Å^3^
*Z* = 2
Mo *K*α radiation
μ = 0.22 mm^−1^
*T* = 293 K
0.22 × 0.21 × 0.15 mm

#### Data collection

Kuma KM-4 four-cricle diffractometer
Absorption correction: analytical (*CrysAlis RED*; Oxford Diffraction, 2008 ▶) *T*_min_ = 0.957, *T*_max_ = 0.970
1955 measured reflections
1821 independent reflections
1439 reflections with *I* > 2σ(*I*)
*R*_int_ = 0.008
3 standard reflections every 200 reflections intensity decay: 0.6%

#### Refinement

*R*[*F* ^2^ > 2σ(*F* ^2^)] = 0.033
*wR*(*F* ^2^) = 0.101
*S* = 1.04
1821 reflections
131 parameters
H atoms treated by a mixture of independent and constrained refinement
Δρ_max_ = 0.42 e Å^−3^
Δρ_min_ = −0.28 e Å^−3^

### 

Data collection: *KM-4 Software* (Kuma, 1996 ▶); cell refinement: *KM-4 Software*; data reduction: *DATAPROC* (Kuma, 2001 ▶); program(s) used to solve structure: *SHELXS97* (Sheldrick, 2008 ▶); program(s) used to refine structure: *SHELXL97* (Sheldrick, 2008 ▶); molecular graphics: *SHELXTL* (Sheldrick, 2008 ▶); software used to prepare material for publication: *SHELXTL*.

## Supplementary Material

Crystal structure: contains datablock(s) I, global. DOI: 10.1107/S1600536812017667/hb6745sup1.cif

Structure factors: contains datablock(s) I. DOI: 10.1107/S1600536812017667/hb6745Isup2.hkl

Additional supplementary materials:  crystallographic information; 3D view; checkCIF report

## Figures and Tables

**Table 1:** Selected bond lengths (Å)

Mg1—O3	2.0212 (10)
Mg1—O1	2.0297 (12)
Mg1—O5	2.0553 (12)

**Table 2:** Hydrogen-bond geometry (Å, °)

*D*—H⋯*A*	*D*—H	H⋯*A*	*D*⋯*A*	*D*—H⋯*A*
N1—H1⋯O2^i^	0.92 (3)	1.85 (3)	2.7711 (16)	175 (2)
N1—H1⋯O1^i^	0.92 (3)	2.58 (2)	3.1446 (16)	119.6 (18)
N2—H3⋯O3^ii^	0.86	2.51	3.1192 (17)	129
N2—H3⋯O5^ii^	0.86	2.55	3.3678 (19)	159
O5—H51⋯O2^iii^	0.86 (3)	1.94 (3)	2.7936 (19)	176 (2)
O5—H52⋯O4^iv^	0.82 (3)	1.90 (3)	2.7108 (16)	167 (3)

Symmetry codes: (i) 
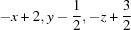
; (ii) 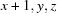; (iii) 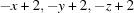; (iv) 
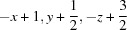
.

**Fig. 1.**
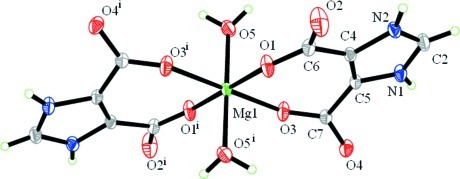
A molecule of the title compound with 50% probability displacement ellipsoids. Symmetry code: (i) -x + 1, -y + 2, -z + 1.

**Fig. 2.**
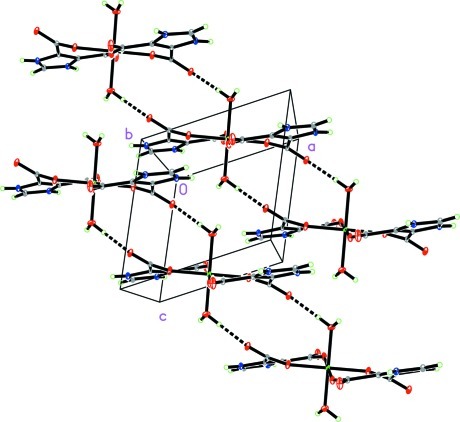
Packing diagram of the structure.

## supplementary crystallographic information

### Comment

The monoclinic structure of the title compound is built of monomeric molecules with Mg^II^ cation in an inversion centre coordinated by two equatorial imidazole-4,5-dicarboxylate ligands which use two carboxylate O atoms, each donated by a different carboxylate group (Fig.1). Hydrogen atom, clearly visible on a Fourier map, attached to the heteroring-N atom maintains charge balance. Two aqua O atoms at axial positions complete a slightly distorted octahedral coordination geometry of the Mg^II^ cation (Table 1). The imidazole ring is almost planar with r.m.s. deviation of 0.0033 (1) Å). The C6/O1/O2 and C7/O3/O4 groups make with the ring dihedral angles of 32.2 (2)° and 8.8 (1)°, respectively. Hydrogen bonds shorter than 3 Å (D···A separation) in which coordinated water O atoms act as donors and carboxylato O atoms in adjacent monomers as acceptors join the monomers into molecular columns. The latter are interconnected by hydrogen bonds in which protonated nitrogen atoms are donors and carboxylato O atoms are acceptors (see, Table 2 and Fig.2). A different coordination mode was reported in the structure of a Ca^II^ monomeric complex with the title ligand: the metal ion, besides four water O atoms, is coordinated by two N,*O* bonding moieties donated by two title ligand molecules. The second carboxylate O atom of this moiety remains protonated and forms a short hydrogen bond of 2.511 (2) Å to the O atom in the adjacent carboxylic group (Starosta *et al.*, 2006). The polymeric structure of a Ba^II^ complex with the title ligand can be described as built of monomeric structural units in which a Ba^II^ ion, like in the Ca^II^ complex, is coordinated by two ligands *via* N,*O* chelating sites. Their second O atoms are protonated and make a short hydrogen bond of 2.495 (2) Å to the O atoms in the adjacent carboxylate group (Starosta *et al.*, 2007).

### Experimental

1 mmol of magnesium(II) nitrate hexahydrate and 2 mmol s of imidazole-4,5-dicarboxylic acid (Aldrich) were dissolved in 20 ml of doubly distilled water, stirred for two hours, closed in a preasure vessel and kept at 363 K for three days. Then, the vessel was slowly cooled to room temperature. The deposited colourless blocks were washed with cold distilled water and dried in the air.

### Refinement

Water and attached to nitrogen hydrogen atoms were located in a difference map and refined isotropically, while the H atom attached to imidazole C atom was located at a calculated position and treated as riding on the parent atom with C—H=0.93 Å and *U*_iso_(H)=1.2*U*_eq_(C).

### Crystal data

**Table:**

[Mg(C_5_H_3_N_2_O_4_)_2_(H_2_O)_2_]	*F*(000) = 380
*M**_r_* = 370.53	*D*_x_ = 1.939 Mg m^−^^3^
Monoclinic, *P*2_1_/*c*	Mo *K*α radiation, λ = 0.71073 Å
Hall symbol: -P 2ybc	Cell parameters from 25 reflections
*a* = 7.2545 (15) Å	θ = 6–15°
*b* = 13.847 (3) Å	µ = 0.22 mm^−^^1^
*c* = 6.9975 (14) Å	*T* = 293 K
β = 115.46 (3)°	Blocks, colourless
*V* = 634.6 (2) Å^3^	0.22 × 0.21 × 0.15 mm
*Z* = 2	

### Data collection

**Table:**

Kuma KM-4 four-cricle diffractometer	1439 reflections with *I* > 2σ(*I*)
Radiation source: fine-focus sealed tube	*R*_int_ = 0.008
Graphite monochromator	θ_max_ = 30.1°, θ_min_ = 2.9°
profile data from ω/2θ scans	*h* = −9→0
Absorption correction: analytical (*CrysAlis RED*; Oxford Diffraction, 2008)	*k* = 0→19
*T*_min_ = 0.957, *T*_max_ = 0.970	*l* = −8→9
1955 measured reflections	3 standard reflections every 200 reflections
1821 independent reflections	intensity decay: 0.6%

### Refinement

**Table:**

Refinement on *F*^2^	Primary atom site location: structure-invariant direct methods
Least-squares matrix: full	Secondary atom site location: difference Fourier map
*R*[*F*^2^ > 2σ(*F*^2^)] = 0.033	Hydrogen site location: inferred from neighbouring sites
*wR*(*F*^2^) = 0.101	H atoms treated by a mixture of independent and constrained refinement
*S* = 1.04	*w* = 1/[σ^2^(*F*_o_^2^) + (0.0578*P*)^2^ + 0.2647*P*] where *P* = (*F*_o_^2^ + 2*F*_c_^2^)/3
1821 reflections	(Δ/σ)_max_ = 0.001
131 parameters	Δρ_max_ = 0.42 e Å^−^^3^
0 restraints	Δρ_min_ = −0.28 e Å^−^^3^

### Special details

**Table:**

Geometry. All e.s.d.'s (except the e.s.d. in the dihedral angle between two l.s. planes) are estimated using the full covariance matrix. The cell e.s.d.'s are taken into account individually in the estimation of e.s.d.'s in distances, angles and torsion angles; correlations between e.s.d.'s in cell parameters are only used when they are defined by crystal symmetry. An approximate (isotropic) treatment of cell e.s.d.'s is used for estimating e.s.d.'s involving l.s. planes.
Refinement. Refinement of *F*^2^ against ALL reflections. The weighted *R*-factor *wR* and goodness of fit *S* are based on *F*^2^, conventional *R*-factors *R* are based on *F*, with *F* set to zero for negative *F*^2^. The threshold expression of *F*^2^ > σ(*F*^2^) is used only for calculating *R*-factors(gt) *etc*. and is not relevant to the choice of reflections for refinement. *R*-factors based on *F*^2^ are statistically about twice as large as those based on *F*, and *R*- factors based on ALL data will be even larger.

### Fractional atomic coordinates and isotropic or equivalent isotropic displacement parameters (Å^2^)

**Table:**

	*x*	*y*	*z*	*U*_iso_*/*U*_eq_	
Mg1	0.5000	1.0000	0.5000	0.01407 (15)	
O2	1.12854 (15)	1.02751 (7)	0.85653 (17)	0.0232 (2)	
O1	0.80572 (15)	1.02280 (7)	0.62178 (18)	0.0232 (2)	
N2	1.18660 (17)	0.84179 (8)	0.72988 (19)	0.0192 (2)	
H3	1.2942	0.8756	0.7557	0.023*	
N1	0.98697 (17)	0.71976 (8)	0.67452 (18)	0.0179 (2)	
O5	0.5127 (2)	1.02541 (9)	0.79479 (18)	0.0302 (3)	
C4	0.99878 (18)	0.87861 (9)	0.69967 (19)	0.0143 (2)	
C5	0.87167 (19)	0.80067 (8)	0.6632 (2)	0.0142 (2)	
C6	0.97418 (19)	0.98431 (9)	0.7277 (2)	0.0145 (2)	
C2	1.1754 (2)	0.74620 (10)	0.7128 (2)	0.0206 (3)	
C7	0.65335 (19)	0.78744 (9)	0.6267 (2)	0.0170 (2)	
H2	1.285 (3)	0.7067 (15)	0.725 (4)	0.035 (6)*	
H51	0.624 (4)	1.0122 (17)	0.904 (4)	0.046 (7)*	
O3	0.54299 (15)	0.86069 (7)	0.59542 (18)	0.0233 (2)	
O4	0.59912 (18)	0.70322 (8)	0.6313 (2)	0.0367 (3)	
H52	0.464 (4)	1.075 (2)	0.820 (4)	0.050 (7)*	
H1	0.942 (4)	0.6569 (18)	0.665 (4)	0.045 (6)*	

### Atomic displacement parameters (Å^2^)

**Table:**

	*U*^11^	*U*^22^	*U*^33^	*U*^12^	*U*^13^	*U*^23^
Mg1	0.0116 (3)	0.0123 (3)	0.0169 (3)	0.0021 (2)	0.0048 (2)	0.0014 (2)
O2	0.0148 (5)	0.0144 (4)	0.0315 (5)	−0.0017 (3)	0.0017 (4)	−0.0022 (4)
O1	0.0128 (5)	0.0144 (4)	0.0346 (6)	0.0016 (3)	0.0028 (4)	0.0040 (4)
N2	0.0121 (5)	0.0174 (5)	0.0267 (6)	0.0012 (4)	0.0070 (4)	0.0018 (4)
N1	0.0163 (5)	0.0119 (5)	0.0233 (5)	0.0027 (4)	0.0064 (4)	0.0002 (4)
O5	0.0307 (6)	0.0379 (6)	0.0189 (5)	0.0178 (5)	0.0077 (4)	−0.0015 (4)
C4	0.0113 (5)	0.0128 (5)	0.0175 (5)	0.0011 (4)	0.0049 (4)	0.0018 (4)
C5	0.0132 (5)	0.0103 (5)	0.0174 (5)	0.0017 (4)	0.0051 (4)	0.0014 (4)
C6	0.0133 (6)	0.0107 (5)	0.0191 (6)	−0.0008 (4)	0.0065 (4)	0.0016 (4)
C2	0.0159 (6)	0.0182 (6)	0.0261 (6)	0.0052 (4)	0.0076 (5)	0.0011 (5)
C7	0.0132 (5)	0.0132 (5)	0.0221 (6)	−0.0005 (4)	0.0053 (4)	0.0031 (4)
O3	0.0156 (5)	0.0152 (4)	0.0393 (6)	0.0037 (3)	0.0120 (4)	0.0066 (4)
O4	0.0217 (5)	0.0142 (5)	0.0715 (9)	−0.0022 (4)	0.0177 (6)	0.0077 (5)

### Geometric parameters (Å, º)

**Table:**

Mg1—O3	2.0212 (10)	N1—C2	1.3266 (18)
Mg1—O3^i^	2.0212 (10)	N1—C5	1.3798 (15)
Mg1—O1^i^	2.0297 (12)	N1—H1	0.92 (3)
Mg1—O1	2.0297 (12)	O5—H51	0.86 (3)
Mg1—O5	2.0553 (12)	O5—H52	0.82 (3)
Mg1—O5^i^	2.0553 (12)	C4—C5	1.3703 (16)
O2—C6	1.2477 (16)	C4—C6	1.4974 (17)
O1—C6	1.2442 (16)	C5—C7	1.5032 (18)
N2—C2	1.3283 (18)	C2—H2	0.94 (2)
N2—C4	1.3833 (16)	C7—O4	1.2355 (16)
N2—H3	0.8600	C7—O3	1.2517 (15)
			
O3—Mg1—O3^i^	180.0	C5—N1—H1	125.1 (16)
O3—Mg1—O1^i^	88.62 (4)	Mg1—O5—H51	118.4 (18)
O3^i^—Mg1—O1^i^	91.38 (4)	Mg1—O5—H52	121.3 (17)
O3—Mg1—O1	91.38 (4)	H51—O5—H52	108 (2)
O3^i^—Mg1—O1	88.62 (4)	C5—C4—N2	106.13 (11)
O1^i^—Mg1—O1	180.0	C5—C4—C6	133.29 (11)
O3—Mg1—O5	84.14 (5)	N2—C4—C6	120.34 (11)
O3^i^—Mg1—O5	95.86 (5)	C4—C5—N1	106.62 (11)
O1^i^—Mg1—O5	90.99 (6)	C4—C5—C7	134.67 (11)
O1—Mg1—O5	89.01 (6)	N1—C5—C7	118.64 (10)
O3—Mg1—O5^i^	95.87 (5)	O1—C6—O2	124.86 (12)
O3^i^—Mg1—O5^i^	84.13 (5)	O1—C6—C4	118.85 (11)
O1^i^—Mg1—O5^i^	89.01 (6)	O2—C6—C4	116.28 (11)
O1—Mg1—O5^i^	90.99 (6)	N1—C2—N2	108.12 (11)
O5—Mg1—O5^i^	180.0	N1—C2—H2	128.0 (13)
C6—O1—Mg1	143.10 (9)	N2—C2—H2	123.9 (13)
C2—N2—C4	109.60 (11)	O4—C7—O3	125.63 (13)
C2—N2—H3	125.2	O4—C7—C5	115.75 (12)
C4—N2—H3	125.2	O3—C7—C5	118.62 (11)
C2—N1—C5	109.51 (11)	C7—O3—Mg1	145.83 (9)
C2—N1—H1	125.3 (15)		

Symmetry code: (i) −*x*+1, −*y*+2, −*z*+1.

### Hydrogen-bond geometry (Å, º)

**Table:**

*D*—H···*A*	*D*—H	H···*A*	*D*···*A*	*D*—H···*A*
N1—H1···O2^ii^	0.92 (3)	1.85 (3)	2.7711 (16)	175 (2)
N1—H1···O1^ii^	0.92 (3)	2.58 (2)	3.1446 (16)	119.6 (18)
N2—H3···O3^iii^	0.86	2.51	3.1192 (17)	129
N2—H3···O5^iii^	0.86	2.55	3.3678 (19)	159
O5—H51···O2^iv^	0.86 (3)	1.94 (3)	2.7936 (19)	176 (2)
O5—H52···O4^v^	0.82 (3)	1.90 (3)	2.7108 (16)	167 (3)

Symmetry codes: (ii) −*x*+2, *y*−1/2, −*z*+3/2; (iii) *x*+1, *y*, *z*; (iv) −*x*+2, −*y*+2, −*z*+2; (v) −*x*+1, *y*+1/2, −*z*+3/2.
